# Global estimated Disability-Adjusted Life-Years (DALYs) of diarrheal diseases: A systematic analysis of data from 28 years of the global burden of disease study

**DOI:** 10.1371/journal.pone.0259077

**Published:** 2021-10-27

**Authors:** Natacha U. Karambizi, Christopher S. McMahan, Carl N. Blue, Lesly A. Temesvari

**Affiliations:** 1 Department of Biological Sciences, Clemson University, Clemson, South Carolina, United States of America; 2 Eukaryotic Pathogens Innovations Center (EPIC), Clemson University, Clemson, South Carolina, United States of America; 3 School of Mathematical and Statistical Sciences, Clemson University, Clemson, South Carolina, United States of America; 4 Department of Graphic Communications, Clemson University, Clemson, South Carolina, United States of America; University of Western Australia, AUSTRALIA

## Abstract

**Background:**

Diarrheal disease (DD)-associated mortality has declined since 1990; however, the incidence of DD has experienced a less-pronounced decrease. Thus, it is important to track progress in managing DD by following loss of healthy years. A disability-adjusted life-year (DALY), which combines data on years-of-life lost (YLL) and years-lived with-disability (YLD), is a metric that can track such a burden.

**Methods and findings:**

Using all 28 years of data in the Global Burden of Diseases, Injuries, and Risk Factors Study (GBD) 2017, we compared DD DALYs among different demographic subsets including sex, age, country, and World Bank (WB) income level. We also evaluated DD DALYs as a function of the socio-demographic index (SDI), a measure of a region’s socio-demographic development. On a global level, DD DALYs have decreased by approximately 85.43% from 1990 to 2017. Incidence and prevalence have decreased by 1.53% and 4.45%, respectively. A dramatic decrease in DD DALYs were observed for WB low-income countries, but not for WB high-income constituents. The temporal decrease in DD DALY rates in WB low-income countries was likely driven by a decrease in YLL. Alternatively, temporal increases in both YLL and YLD may have contributed to the apparent lack of progress in WB high-income countries. Regardless of WB income classification, children under the age of five and the elderly were the most vulnerable to DD. In nearly every year from 1990 to 2017, DD DALYs for females were higher than those for males in WB high-income regions, but lower than those for males in WB low-income constituents. The reason for these differences is not known. We also observed that the rate of DD DALYs was highly correlated to SDI regardless of WB income classification.

**Conclusions:**

To the best of our knowledge, this is the only temporal study of DD DALYs that encompasses all 28 years of data available from the GBD. Overall, our analyses show that temporal reductions in DD DALYs are not equivalent across regions, sexes and age groups. Therefore, careful attention to local and demography-specific risk factors will be necessary to tailor solutions in region- and demography-specific manners.

## Introduction

Diarrheal diseases (DD) have long been distinguished as a serious global health problem, particularly in developing countries [1–3]. Acute diarrhea is typically caused by infection with enteric pathogens, such as viruses (e.g., norovirus [4], rotavirus [5]), bacteria (e.g., *Campylobacter* [6], *Escherichia coli* [7], *Salmonella* [8], *Shigella* [9]), fungi [10], or parasites (e.g., *Entamoeba h*is*tolytica* [11], *Giardia lamblia* [12], *Cryptosporidium enterit*is [13]). On the other hand, chronic diarrhea can have non-communicable derivations such as food allergies and intolerances, celiac disease, Crohn’s disease, ulcerative colitis, or long-term use of medicines, such as antibiotics, antacids, or other drugs [14–17].

According to World Health Organization (WHO) Global Health Observatory (GHO) data, in 2016, diarrhea was the 9^th^ leading cause of mortality, responsible for more than 1.3 million global deaths [18]. Many factors including age, environment, and sex can impact the severity of disease. For example, the impact of DD on children is substantial. WHO GHO data show that in 2016, DD ranked fourth among all causes of mortality in children younger than five [18]. Multiple reports have explored reasons for this burden in children [19–21]. Furthermore, there is increasing evidence that shows that children, who suffer from multiple bouts of diarrhea before the age of two, experience poor physical growth [22], poor cognitive development [23, 24], and are at higher risk for the development of type 2 diabetes, metabolic syndrome, obesity, and related co-morbidities [25, 26]. Institutionalization of aging populations further challenges the ability to limit DD. According to the United Nations, individuals older than 60 years of age will grow from 962.3 million in 2017 to 2080.5 million in 2050 [27]. This growth will occur mainly in Africa, for which it is estimated that individuals over the age of 60 will increase by 230% over this time period.

Location is another factor that has bearing on the number of DD deaths. An important framework for the management of DD is that such maladies occur more frequently in low-income countries. Lack of infrastructure and resources in many low-income countries may pose substantial challenges to adequately addressing DD in these areas [28]. For example, in many low-income countries, lack of sanitary facilities and hygienic practices, fly infestations, and regular consumption of street food constitute some of the leading causes of DD [29, 30]. Evidence has shown that World Bank (WB) low-income regions also have higher numbers of marginalized populations, who are the most vulnerable. For example, 87% of DD deaths occurred in South Asia and sub-Saharan Africa in 2015 [21].

Slightly more than half (52.23%) of all diarrheal deaths in 2017 were in women [31]. Cultural factors may influence a disparity among sexes. For instance, in some sub-Saharan countries experiencing instability and violence, many women have reverted to using unclean forms of sanitation, such as bucket toilets. This practice is driven by fear of physical and sexual violence that comes with using public sanitation facilities [32, 33]. Other research suggests that the sanitation practices used by women may be influenced by their fear of contracting infections from unclean facilities [28, 34]. On the other hand, researchers, who were studying children under the age of five in northern Sudan, found that boys were more likely to contract DD than girls, perhaps because boys were given more freedom to play outside of the home than girls and thus, were more likely to stray into unsanitary environments [35].

Based on mortality, overall global health has improved tremendously as life-expectancy has almost doubled since the 1950s [36]. Much of this progress is the result of improvements that have led to a reduction of child mortality rates. For example, since 1990, overall mortality (all causes) for children under five has declined by 57%, and mortality due to diarrheal causes has declined by 68% [31]. Despite a drop in diarrhea-related mortality, the incidence of DD in this same age group, has experienced a less-pronounced declivity [31], as it has decreased by only 24% during the same time period.

Mortality indicators are valuable; however, they do not furnish all the data that is essential for measuring the health of a population, or for evaluating interventions. For example, a mortality statistic does not disclose the consequences of illness prior to recovery or death. Summary measures, which include data on morbidity as well as mortality, capture the true burden of disease. One such measure is a disability-adjusted life-year (DALY). First introduced in the World Development Report 1993 [37] and the Disease Control Priorities Review [38], it incorporates both current and long-term burden and incorporates morbidity as years-of-life-lost (YLL) because of premature mortality, as well as years-lived-with-disability (YLD). One DALY equals the sum of the YLL and YLD and is comparable to one lost year of “healthy” life. The sum of DALYs across a population expresses the burden of disease caused by a gap between current health status and optimal health. Given that (i) DD-associated deaths and incidence have declined at different rates, (ii) DD in childhood have long-term impacts on physical health and cognition, and (iii) elderly populations will continue to increase, it is important to also track progress in managing DD by following loss of healthy years, using a summary measure.

Therefore, the goal of this study was to examine the burden of DD from 1990 to 2017 based on DALY data from the Global Burden of Diseases, Injuries, and Risk Factors Study 2017 (GBD2017) [31]. Specifically, we performed a multi-year analysis of DD DALYs associated with DD by WB income region, age, and sex. We also examined the relationship between DD DALYs and the sociodemographic index (SDI). SDI was developed by the GBD Project 2015 and is a summary measure of a country’s or geographic area’s socio-demographic development. The statistic is comprised of data on education level, average income per person, and total fertility rate (TFR). The SDI measure is expressed on a scale of 0 to 1. A zero SDI value indicates the lowest income per capita, the lowest educational achievement, and highest TFR, while an SDI value of one indicates the highest income per capita, the highest educational realization, and the lowest TFR.

Several previous studies have provided robust information about DD DALYs by analyzing data from single years, for single etiological agents, or for one age group [21, 39–42]. To the best of our knowledge, this is the only protensive study of DD DALYs that encompasses all ages and causes for all 28 years of data available from the GBD. To the best of our knowledge, this is also the only study to examine the relationship between DD DALYs and sociodemographic status.

## Materials and methods

### Overview of data sets

The GBD project (1990-present) is a long-standing global endeavor that provides wide-ranging, global-level, assessments of data on human health. Each year, a consortium of project collaborators track mortality and morbidity associated with both communicable and non-communicable diseases. The data in the GBD2017 are extracted from 68,781 data sources and are reported by region, age, and sex, which allows for a variety of comparisons by time, sex, population and other demographic groupings [31]. GBD2017 data sets encompass 359 diseases, with 3484 sequelae and 84 risk factors for each of 195 countries and territories. The first data set (for the initial year 1990) was published in the World Development Report 1993 [37]. This international consortium is managed by the Institute for Health Metrics and Evaluation (IHME; Seattle, WA).

### Analysis of data

Using multi-year GBD data, we analyzed the impact of DD using Microsoft Office Excel 2019 (Microsoft, Redmond, WA). Specifically, we compared the disability-adjusted life-year (DALY) metric due to DD among different demographic subsets including sex, age, country, and WB high- or low-income regions. WB high-income countries are defined as those with a gross national income (GNI) per capita greater than or equal to US $12,376 and WB low-income countries are defined as those with a GNI per capita less than or equal to US $1,025 [43]. In all cases, except when comparing DD DALYs by age, we used age-standardized data, which enabled comparison across populations with different age demographics. All data estimates in the GDB2017 study were listed with 95% uncertainty intervals (UIs).

We also evaluated DD DALYs as a function of the socio-demographic index (SDI). This evaluation was done with global data, as well as with WB high- and WB low-income country data, to determine which possessed more volatile trends. To accomplish this, the following penalized spline regression model was posited

Y=f(x)+ϵ,

where *Y* denotes DD DALYs per 100,000, *x* denotes average SDI, *f*(∙) is an unknown function, and *ϵ* is the usual error term. To examine volatility while also acknowledging the non-linear nature of the problem, a natural estimator would take the form ∫{*f*^(1)^(*x*)}^2^*dx* This estimator examines the squared rate of change in *f*(∙) across its entire support and thus serves to summarize the total volatility. To retain modeling flexibility, we viewed *f*(∙) as an unknown function without a specified form. This assumption translates to *f*(∙) being an infinite dimensional parameter. To reduce the dimensionality of the problem, we approximated this parameter via B-splines [44]; i.e., as

$$f(x)=\gamma_{0}+\sum_{j=1}^{J}B_{j}(x)\gamma_{j},$$

where *B*_*j*_(*x*) is a B-spline basis function, *γ*_*j*_ is the corresponding spline coefficient, for *j* = 1,…, *J*, and *γ*_0_ is an intercept parameter. Each of the basis functions were uniquely determined once a knot sequence and degree were specified [44]. In this application we used a knot set consisting of 3 interior knots (placed at equally spaced quantiles) and specified the degree to be 3. To smoothly estimate the functional coefficient and to avoid overfitting, we used a regularizing penalty which encourages the smoothing of our functional estimator. This process yields the following penalized estimator

$${\hat{\gamma }}_{\lambda }{=argmin}_{\gamma }\sum_{i=1}^{m}{\left\{Y_{i}-f(x_{i})\right\}}^{2}+\lambda \int {\left\{f^{\left(1\right)}(x)\right\}}^{2}dx,$$

where ***γ*** = (*γ*_0_,*γ*_1_,…,*γ*_*J*_)′ is the collection of unknown parameters, *λ* is a penalty parameter, ${\hat{\gamma }}_{\lambda }$ is a penalty parameter specific estimator of ***γ***, and *f*^(1)^(*x*) is the first derivative of *f*(*x*). To choose the penalty parameter we first noted that

$${\hat{\gamma }}_{\lambda }={\left\{B^{\prime }B+R^{*}(\lambda )\right\}}^{-1}B^{\prime }Y,$$

where $Y=(Y_{1},\ldots ,Y_{m})\prime ,\;B=(B_{1}^{\prime },\ldots ,B_{m}^{\prime })\prime ,\;B_{i}=(1,B_{1}(x_{i}),\ldots ,B_{J}(x_{i}))\prime$, and R*(*λ*) is a (*J*+1)×(*J*+1) matrix whose first row and column are all zeros and whose remaining entries are given by ${R^{*}(\lambda )}_{jj^{\prime }}=\lambda B_{j-1}^{\left(1\right)}(x)B_{j^{\prime }-1}^{\left(1\right)}(x)$. From this form it was easy to identify the so-called smoother matrix as ***S***_*λ*_ = ***B***{***B***′***B***+R*(*λ*)}^−1^***B***′. This matrix was used to quantify “degrees of freedom” of the model under a certain penalty parameter. In particular, we compute the degrees of freedom as *df*(*λ*) = *tr*(***S***_*λ*_). This value was then used to compute the Schwartz Bayesian Information Criterion (BIC). Using BIC to guide the selection of the optimal penalty parameter, denoted by say λ*, we then assessed volatility of *f*(∙) by computing ${\hat{\gamma }}_{\lambda^{*}}\prime R^{*}(\lambda^{*}){\hat{\gamma }}_{\lambda^{*}}$ which is an estimate of the ∫{*f*^(1)^(*x*)}^2^*dx*. To quantify uncertainty in this estimate, we constructed 95% bootstrap confidence intervals for this parameter.

### Data visualization

Data were visualized using the IHME GBD Compare Data Visualization tool [45].

### Ethics statement

This study was based on secondary databases, which are publicly available, without identification of individual data.

## Results

### Summary of global DALYs due to diarrheal disease

The S1 Table summarizes the global estimates for DD DALYs, incidence, and prevalence for DD from 1990 and 2017. In 1990, total global DD DALYs were estimated at 178,669,278.77 (95% UI: 154,235,572.29–203,868,117.47) with an age-standardized DD DALY rate of 3227.01 per 100,000 people. In 2017, DD DALYs were estimated at 81,039,363.85 (95% UI:70,120,051.79–97,233,423.45); and a markedly lower age-standardized DD DALY rate of 1262.76 per 100,000 population globally. Thus, diarrheal DD DALY rates have decreased by approximately 85.43% from 1990 to 2017.

A less dramatic decrease in DD incidence and prevalence rates is evident. DD incidence and prevalence rates have decreased only by 1.53% and 4.45%, respectively, from 1990 to 2017 (S1 Table). The DD incidence rate was estimated to be 85,808.8262 per 100,000 (95% UI: 78,417.423–93,386.4644–135,836,824.13) in 1990, and 84,415.44027 per 100,000 (95% UI: 77,720.046–91,536.162) in 2017. DD prevalence rate was estimated to be 1,333.944262 (95% UI: 1,232.43–1,438.42) in 1990 and 91,269.775151 per 100,000 (95% UI: 1,180.95–1,364.57) in 2017.

### Global diarrheal disease DALYs by World Bank (WB) economic region

**Fig 1:**
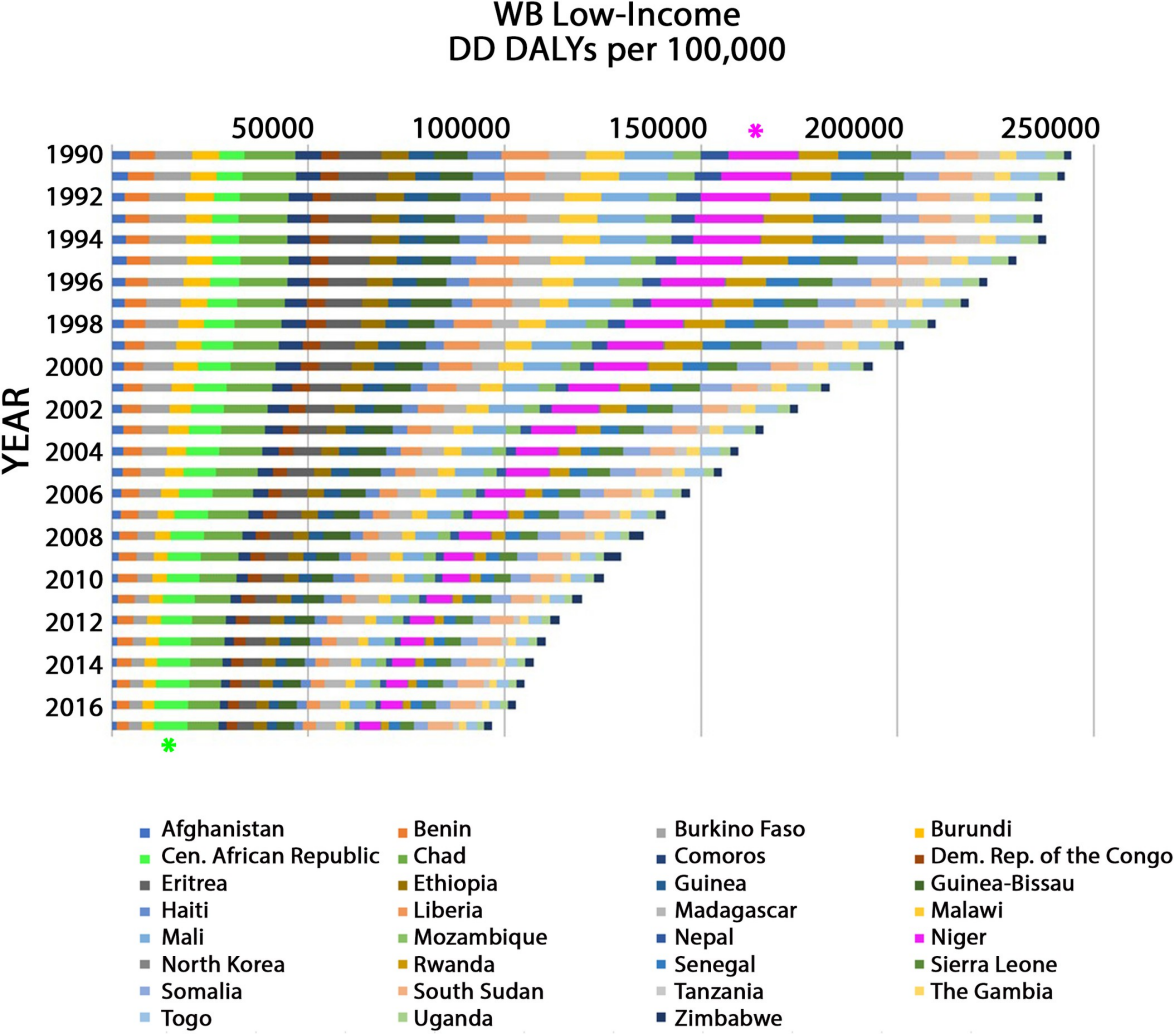
Age-standardized DD DALY rates from 1990 to 2017 in WB low-income constituents. For each year (bar), the total DD DALY rate represents the sum of individual country DD DALY rates given in alphabetical order from left to right as follows: Afghanistan, Benin, Burkina Faso, Burundi, Central (Cen.) African Republic, Chad, Comoros, Democratic (Dem.) Republic (Rep.) of Congo, Eritrea, Ethiopia, Guinea, Guinea-Bissau, Haiti, Liberia, Madagascar, Malawi, Mali, Mozambique, Nepal, Niger, North Korea, Rwanda, Senegal, Sierra Leone, Somalia, South Sudan, Tanzania, The Gambia, Togo, Uganda and Zimbabwe. Asterisks identify the countries with the highest number of DD DALYs per 100,000 in 1990 (Niger; magenta asterisk) or in 2017 (Central African Republic; green asterisk).

**Fig 2:**
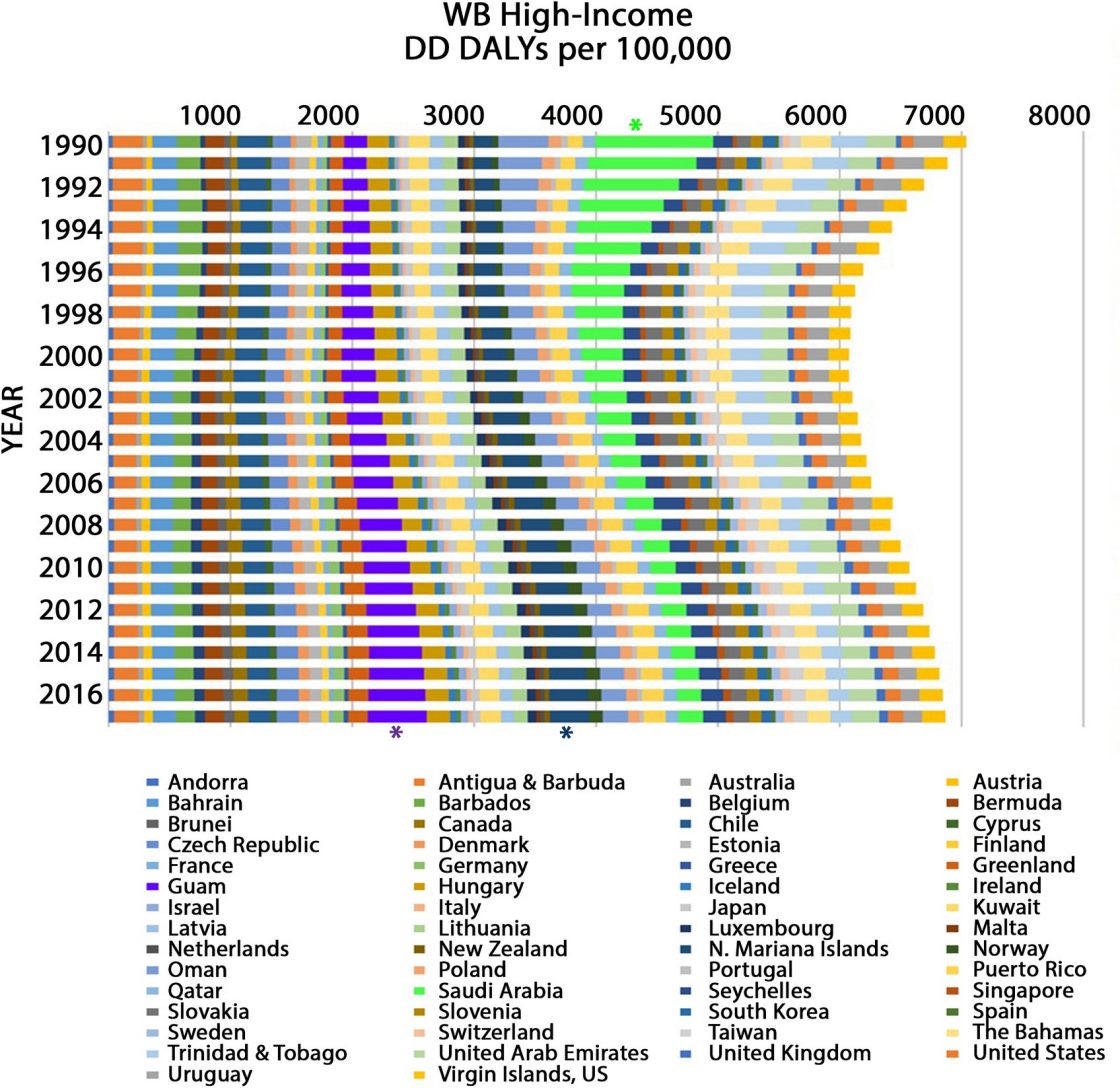
Age-standardized DD DALY rates from 1990 to 2017 in WB high-income constituents. For each year (bar), the total DD DALY rate represents the sum of individual country DD DALY rates given in alphabetical order from left to right as follows: Andorra, Antigua and Barbuda, Australia, Austria, Bahrain, Barbados, Belgium, Bermuda, Brunei, Canada, Chile, Cyprus, Czech Republic, Denmark, Estonia, Finland, France, Germany, Greece, Greenland, Guam, Hungary, Iceland, Ireland, Israel, Italy, Japan, Kuwait, Latvia, Lithuania, Luxembourg, Malta, Netherlands, New Zealand, Northern (N.) Mariana Islands, Norway, Oman, Poland, Portugal, Puerto Rico, Qatar, Saudi Arabia, Seychelles, Singapore, Slovakia, Slovenia, South Korea, Spain, Sweden, Switzerland, Taiwan, The Bahamas, Trinidad and Tobago, United Arab Emirates, United Kingdom, United States, Uruguay, and Virgin Islands. Asterisks identify the countries with the highest number of DD DALYs per 100,000 in 1990 (Saudi Arabia; green asterisk) or in 2017 (Guam; purple asterisk; Northern Mariana Islands; blue asterisk).

Although global DD DALYs decreased from 1990 to 2017 (S1 Table), we sought to determine if different WB economic regions varied in their progress in the management of DD. The collective rate of DD DALYs, for all WB low-income regions combined, has been in steady decline (Fig 1). DD DALY rates per 100,000 in 2017 (100,905.15) were 58.69% of that in 1990 (244,252.09). This is a marked decrease in the burden of DD over 28 years. In 1990, in WB-low-income countries, DD DALYs per 100,000 were highest in Niger with an estimate of 17,633.63 (95% UI: 12,873.65–22,679.68) (magenta asterisk, Fig 1). In 2017, the DD DALYs per 100,000 were highest in Central African Republic with an estimate of 8,590.04 (95% UI: 5,449.51–12,230.82) (green asterisk, Fig 1). In fact, DD DALY rates for Central African Republic, in 2017 (8,590.04), were 34.21% greater than that in 1990 (6,400.68). For all years from 1990–2017 North Korea had the lowest number of DD DALYs per 100,000 (Fig 1). Unsurprisingly, there were substantially more total DD DALYs per 100,000 in WB low-income regions (Fig 1) than in high-income regions (Fig 2) with a ratio of 34.9:1 in 1990 and 14.8:1 in 2017.

In 1990, in WB high-income regions, Saudi Arabia exhibited the highest DD DALYs per 100,000 with an estimate of 962.35 (95% UI 662.02–1374.01) (green asterisk, Fig 2), and the Netherlands had the lowest DD DALYs per 100,000 with an estimate of 16.52 (95% UI: 12.27–21.60). In 2017, the US territories of Guam (purple asterisk, Fig 2) and the Northern Mariana Islands (blue asterisk, Fig 2) were among those constituents with the highest DD DALYs per 100,000, with estimates of 483.37 (95% UI: 335.04–663.90) and 326.23 (95% UI: 230.68–443.61), respectively. In 2017, Malta had the lowest DD DALYs per 100,000 with an estimate of 25.90 (95% UI: 19.58–33.28). Furthermore, there was an interesting temporal trend in WB high-income countries. Unlike the steady annual decline seen in WB low-income countries, DD DALY rates in WB high-income countries decreased from 1990 to 1998, after which they increased almost every year up to 2017 (Fig 2). Thus, in WB high-income regions, there was only a 2.49% cumulative decrease in DD DALYs per 100,000 from 1990 to 2017. In particular, DD DALYs per 100,000 escalated each year from 1990 to 2017 in the US Territories of Guam (+5.67% per year on average), and the Northern Mariana Islands (+4.01% per year on average) and nearly each year from 1990 to 2017 in Denmark (+3.06% per year on average), Germany (+3.78% per year on average), and the US Territory of Puerto Rico (+1.62% per year on average).

**Fig 3:**
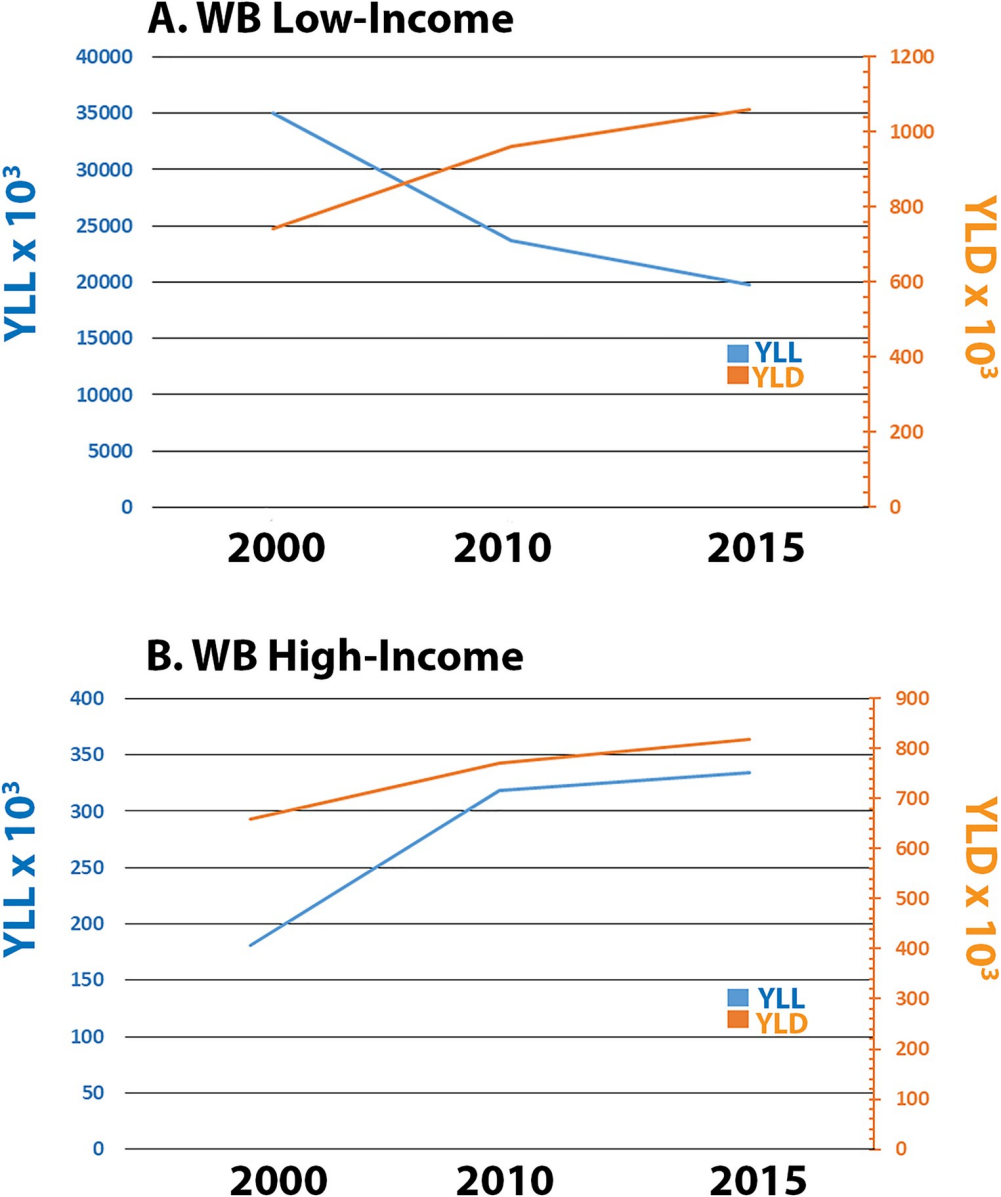
Years-of-life-lost (YLL) and years-lived-with-disability (YLD) due to DD. YLL and YLD data are available from GBD2017 for the years 2000, 2010 and 2015. DD YLLs (x 10^3^) and YLDs (x 10^3^) for WB low-income (A) and WB high-income (B) countries by year are presented.

Since DD DALYs are a metric composed of YLL and YLD, we sought to determine the contribution of each of these measures to the temporal patterns of DD DALYs observed for WB low-income and high-income constituents. YLL and YLD data are readily available for some of the years over the 28-year study period (e.g., 2000, 2010, 2015). In WB low-income regions, YLL exhibited a progressive decline, while YLD exhibited a progressive increase over time (Fig 3A). Thus the temporal decrease in DD DALYs in WB low-income countries is primarily driven by a decrease in YLL. Alternatively, in WB high-income countries, increases in both YLL and YLD (Fig 3B) were observed over the study period, both of which may be contributing to the apparent lack of progress in WB high-income constituents.

Along with WB income classification, the sociodemographic index (SDI) provides a measure of the developmental progress of a region. The S1 Table also includes the annual socio-demographic index (SDI) as reported in the GBD2017 results. SDI values increased from 0.52 in 1990 to 0.65 in 2017, indicating that there has been global economic progress overall.

**Fig 4:**
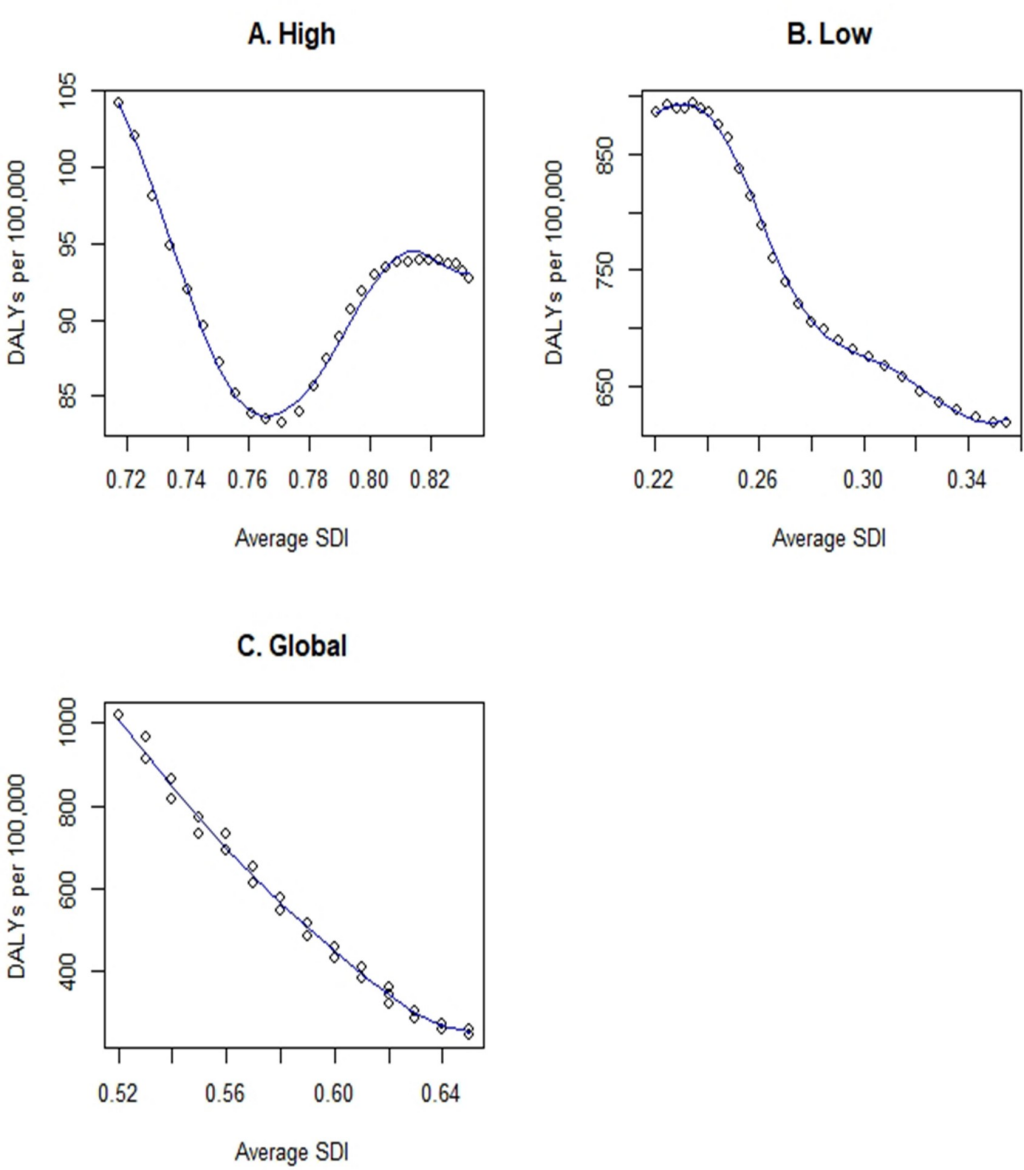
The relationship between DD DALYs and sociodemographic index (SDI) for WB high-income (A), WB low-income (B) and global data combined (C). Each data point represents a single year from 1990–2017 and represents totaled DD DALYs per 100,000 for all WB high-income countries (A), WB low-income countries (B), or globally (C). The volatility statistic had a value of 13,254.65 for WB high-income regions (95% CI: 7,970.436, 13,837.297), 985,066.7 for WB low-income regions (95% CI: 897,082.9, 1,074,332.5), and 4,754,435.2 globally (95% CI: 3,415,048.0, 5,111,699.1).

We plotted global DD DALY rates from 1990 to 2017 against the annual WB high-income SDI (Fig 4A), WB low-income SDI (Fig 4B), and global SDI (Fig 4C). These analyses reveal a highly non-linear relationship in the WB high-income regions, where SDI values are highest and DD DALYs lowest. Looking at the curve, one will note that it closely imitates the trend we saw in Fig 2, where there was at first a decrease in the number of DD DALYs from 1990 to 1998, after which the number of DD DALYs started to increase. In contrast, DD DALY rates in WB low-income regions and globally had more of a linear relationship with SDI value. As the SDI increases, DD DALY rates per 100,000 decrease over time. The penalized spline regression model was fit to these data. Fig 4 depicts the resulting model fits. As a measure of overall change, the volatility statistic was computed for each. This measure yielded a value of 13,254.65 for high-income regions (95% CI: 7,970.436, 13,837.297), 985,066.7 for low-income regions (95% CI: 897,082.9, 1,074,332.5), and 4,754,435.2 globally (95% CI: 3,415,048.0, 5,111,699.1). This analysis reveals that the rate of change (i.e., volatility) was the smallest for high-income regions. In other words, the relationship between SDI values and DD DALYs was the flattest.

### Global diarrheal diseases DALYs by sex

To determine if DD DALYs in males and females were differentially contributing to the overall temporal patterns of DD DALYs, we compared age-standardized DD DALY rates per 100,000 by sex in both economic regions.

**Fig 5:**
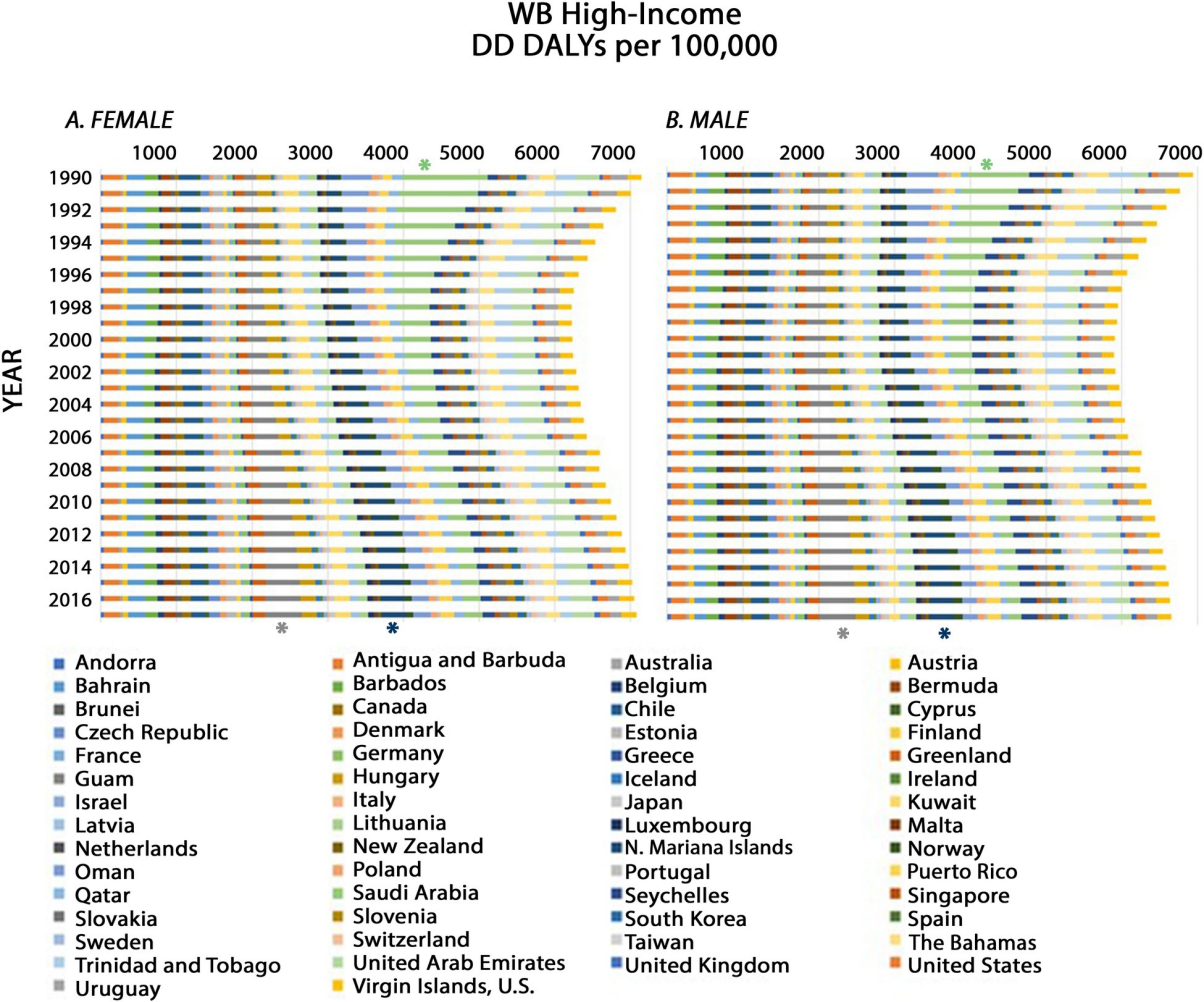
Age-standardized DD DALY rates from 1990 to 2017 in WB high-income constituents for females (A) and males (B). For each year (bar), the DD DALY rate per 100,000 represents the sum of individual country DD DALY rates given in alphabetical order from left to right as follows: Andorra, Antigua and Barbuda, Australia, Austria, Bahrain, Barbados, Belgium, Bermuda, Brunei, Canada, Chile, Cyprus, Czech Republic, Denmark, Estonia, Finland, France, Germany, Greece, Greenland, Guam, Hungary, Iceland, Ireland, Israel, Italy, Japan, Kuwait, Latvia, Lithuania, Luxembourg, Malta, Netherlands, New Zealand, Northern (N.) Mariana Islands, Norway, Oman, Poland, Portugal, Puerto Rico, Qatar, Saudi Arabia, Seychelles, Singapore, Slovakia, Slovenia, South Korea, Spain, Sweden, Switzerland, Taiwan, The Bahamas, Trinidad and Tobago, United Arab Emirates, United Kingdom, United States, Uruguay, and Virgin Islands. Asterisks identify the countries with the highest number of DD DALYs per 100,000 in 1990 (Saudi Arabia; green asterisk) or in 2017 (Guam; grey asterisk; Northern Mariana Islands; blue asterisk).

The sex-specific temporal pattern of DD DALY rates in WB high-income countries (Fig 5) followed that for both sexes combined (Fig 2). Specifically, there was an apparent decline in DD DALYs per 100,000 from 1990 to approximately 1998, after which there was an increase almost every year up to 2017. Once again, in 1990, Saudi Arabia reported the highest standardized DD DALYs for both females and males with estimates of 1,126.68 (95% UI: 725.03–1,768.16), and 813.99 (95% UI: 505.30–1268.25), respectively (green asterisk, Fig 5). Likewise, in 2017, Guam (grey asterisk, Fig 5) and the Northern Mariana Islands (blue asterisk, Fig 5) were among the constituents that reported the highest standardized DD DALYs for both males and females. The 2017 Guam age-standardized estimates for males and females, were 481.44 (95% UI: 331.90–669.58) and 487.36 (95% UI: 333.81–671.90), respectively, and the 2017 Northern Mariana Island estimates, for males and females, were 324.57 (95% UI: 226.01–439.97) and 327.65 (95% UI: 227.92–449.68), respectively.

**Fig 6:**
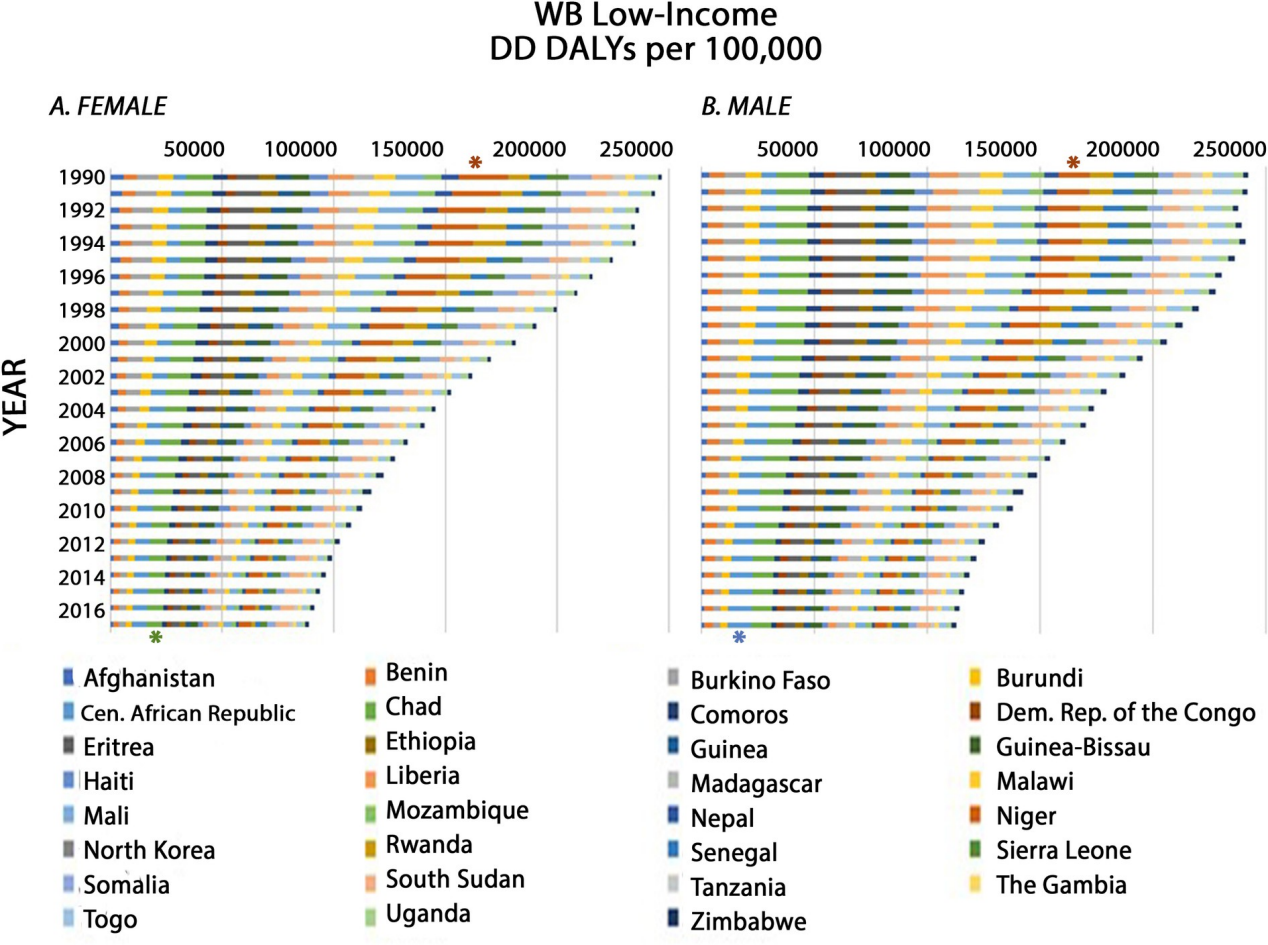
Age-standardized DD DALY rates from 1990 to 2017 in WB low-income constituents for females (A) and males (B). For each year (bar), the DD DALY rate per 100,000 represents the sum of individual country DD DALY rates given in alphabetical order from left to right as follows: Afghanistan, Benin, Burkina Faso, Burundi, Central (Cen.) African Republic, Chad, Comoros, Democratic (Dem.) Republic (Rep.) of Congo, Eritrea, Ethiopia, Guinea, Guinea-Bissau, Haiti, Liberia, Madagascar, Malawi, Mali, Mozambique, Nepal, Niger, North Korea, Rwanda, Senegal, Sierra Leone, Somalia, South Sudan, Tanzania, Togo, The Gambia, Uganda and Zimbabwe. Brown asterisks identify data from Niger, which had the highest number of DD DALYs per 100,000 for both females (value = 21,842.85 (95% UI: 14,124.44566–29,316.44)) and males (value = 13,595.21 (95% UI: 8,862.79–19,531.25) in 1990. The blue asterisk identifies data from Central African Republic, which had the highest number of DD DALYs per 100,000 for males (value = 10,497.14 (95% UI: 5,794.68–16,518.80)) and the green asterisk identifies Chad, which had the highest number of DD DALYs per 100,000 for females (value = 6,963.74 (95% UI: 5,098.99–9,369.92)) in 2017.

The sex-specific temporal pattern of DD DALY rates in WB low-income countries (Fig 6) was also like that for both sexes combined (Fig 1). In 1990 Niger once again, had the highest DD DALY rates for both females and males at 21,842.85 (95% UI: 14,124.44566–29,316.44) and 13,595.21 (95% UI: 8,862.79–19,531.25), respectively (brown asterisk, Fig 6). In 2017 Central African Republic exhibited the highest DD DALY rates per 100,000 for males with a value of 10,497.14 (95% UI: 5,794.68–16,518.80) (blue asterisk, Fig 6), and Chad had the highest age-standardized rates for females with 6,963.74 (95% UI: 5,098.99–9,369.92).

Interestingly, in each year from 1990 to 2017, standardized DD DALYs for females in WB high-income regions slightly exceeded those for males (S1 Fig). However, in WB low-income constituents the opposite trend was observed. Specifically, standardized DD DALYs for males slightly exceeded those for females in most years from 1990 to 2017 (S1 Fig).

To further examine this pattern country-by-country, and to determine if the trend was observable at the beginning and end of data collection (i.e., 1990 versus 2017), we plotted the data side-by-side for each sex by country (S2 and S3 Figs). Although total standardized DD DALYs for females exceeded those for males in WB high-income countries (S1 Fig), the pattern was not observed for every country (S2 Fig). In 1990 50% of WB high-income nations exhibited higher standardized DD DALYs per 100,000 for females than for males, and in 2017 43% of WB high-income nations exhibited higher standardized DD DALYs per 100,000 for females than for males. In 1990, Saudi Arabia exhibited the highest standardized DD DALY rates for both males and females. In Saudi Arabia, the rate of DD DALYs in females was 35.79% higher than that in males. In 2017, Guam exhibited the highest standardized DD DALY rates for both males and females. However, the gap between the sexes was considerably smaller; there was a 1.23% difference in DD DALY rates between females and males (S2 Fig).

In 1990, 35.48% of WB low-income nations exhibited higher standardized DD DALYs per 100,000 for males than for females, and 2017 none of the WB low-income nations exhibited higher standardized DD DALYs per 100,000 for males than for females (S3 Fig). In 1990 Niger exhibited the highest standardized DD DALYs per 100,000 for both males and females. In Niger, DD DALYs per 100,000 were 60.67% higher in females than in males. However, in 2017, Chad exhibited the highest standardized DD DALYs for females, whereas Central African Republic exhibited the highest standardized DD DALYs for males.

### Global diarrheal diseases DALYs by age

**Fig 7:**
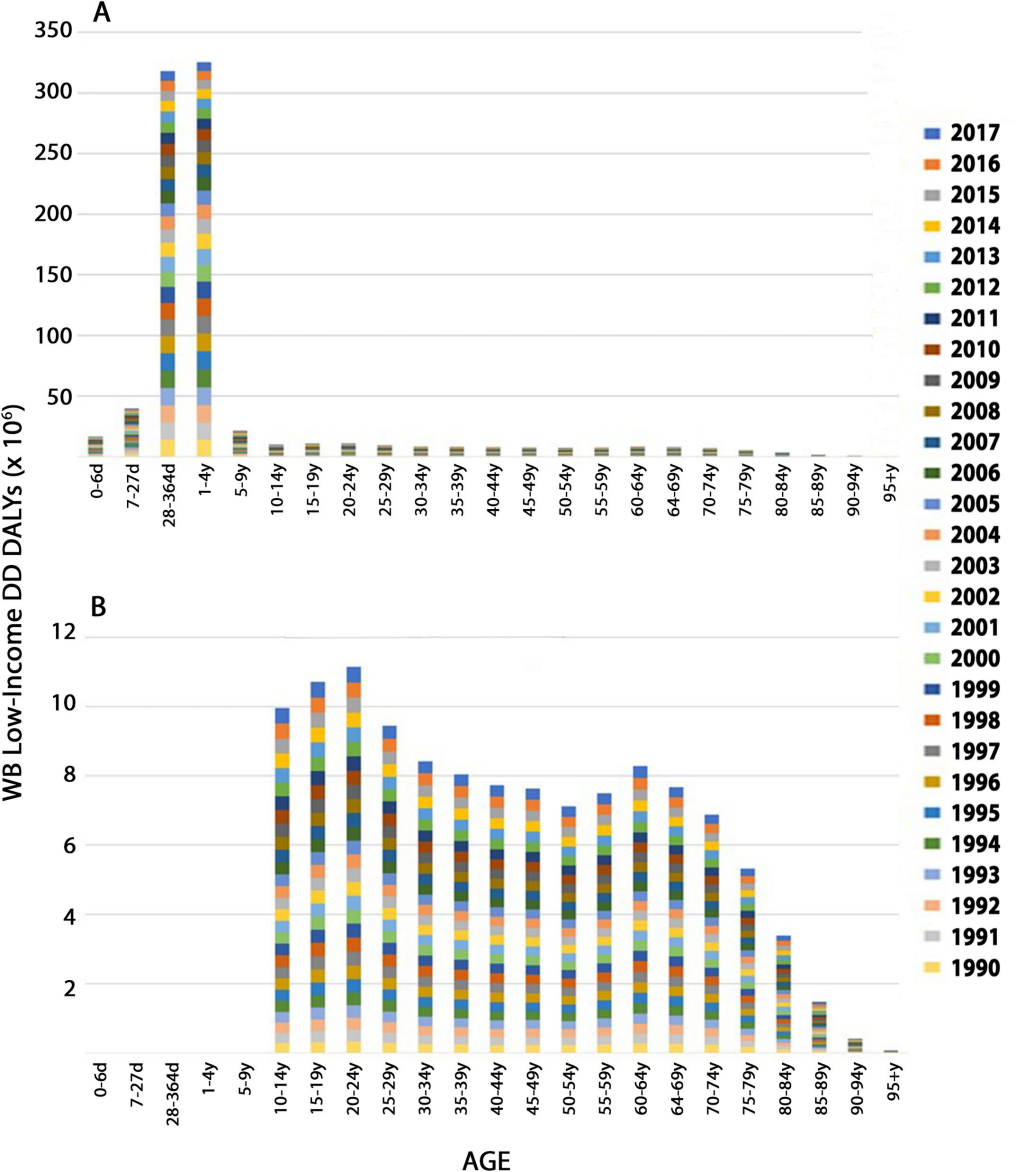
The relationship between age-standardized DD DALY and age for WB low-income countries. Age is given in days (d) or years (y). The data are plotted with (A) and without (B) data from the under 10-year-old age classes. For each age class (bar), the sum of annual DD DALYs from 1990–2017 are presented.

It is well-established that children under five are particularly vulnerable to DD [46]. When considering DD DALY counts, it was not surprising that in WB low-income countries, the 28 to 364 days and 1 to 4 years age categories emerged as the most vulnerable of the demographic groups (Fig 7A). In 1990, there were a total of 13,949,996.98 DD DALYs (95% UI: 1,172,1297.30–1,622,0597.90) for the 28- to 364-day age group and a total of 13,905,595.71 DD DALYs (95% UI: 10,921,075.73–16,896,492.69) for the 1- to 4-year age group. In 2017, there were a total of 8,170,515.87 DD DALYs (95% UI: 7,028,902.56–9,511,204.72) for the 28- to 364-day age group in 2017 and a total of 7,356,749.81 DD DALYs (95% UI: 6,073,493.12–8,761,415.09) in 2017 for the 1- to 4-year age group.

**Fig 8:**
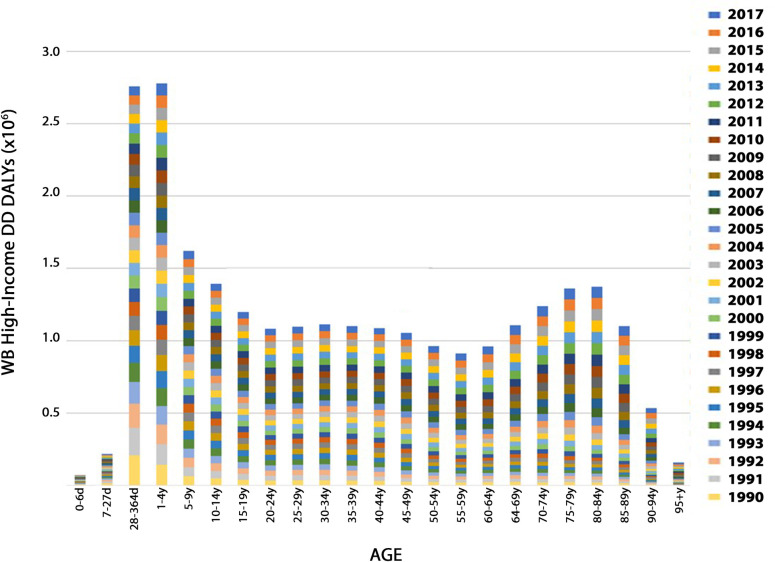
The relationship between age-standardized DD DALY and age for WB high-income countries. Age is given in days (d) or years (y). For each age class (bar), the sum of annual DD DALYs from 1990–2017 are presented.

To further analyze DD DALYs by age in WB low-income countries, we re-scaled the data by removing information for the first ten years (Fig 7B). Two additional peaks in standardized DD DALYs were observed: one in the 20- to 24-year age group, and one in the 60 to 64-year age group. The apparent increase in DD DALYs for 20- to 24-year-old individuals has not been the focus of previous studies. As in WB low-income countries, children under 5 years in WB high-income constituents seemed to be the most vulnerable of demographic age groups (Fig 8). In WB high-income countries, infants 28 to 364 days old were the most vulnerable to DD with a total of 206,133.79 (95% UI: 145,965.76–292,139.86) DD DALYs in 1990 (Fig 8). However, in 2017 the most vulnerable group was the 1 to 4-year-olds with a total of 83,578.53 (95% UI: 53,231.443–124,013.45) DD DALYs (Fig 8). A second peak in DD DALYs was seen between the ages of 70 and 89 years old. This peak occurs in an older population in WB high-income countries (70 to 89 years of age) than that in WB low-income regions (60 to 64 years of age) (Fig 7B). A slight peak of DD DALYs was also seen in the 30 to 34-year old age group.

**Fig 9:**
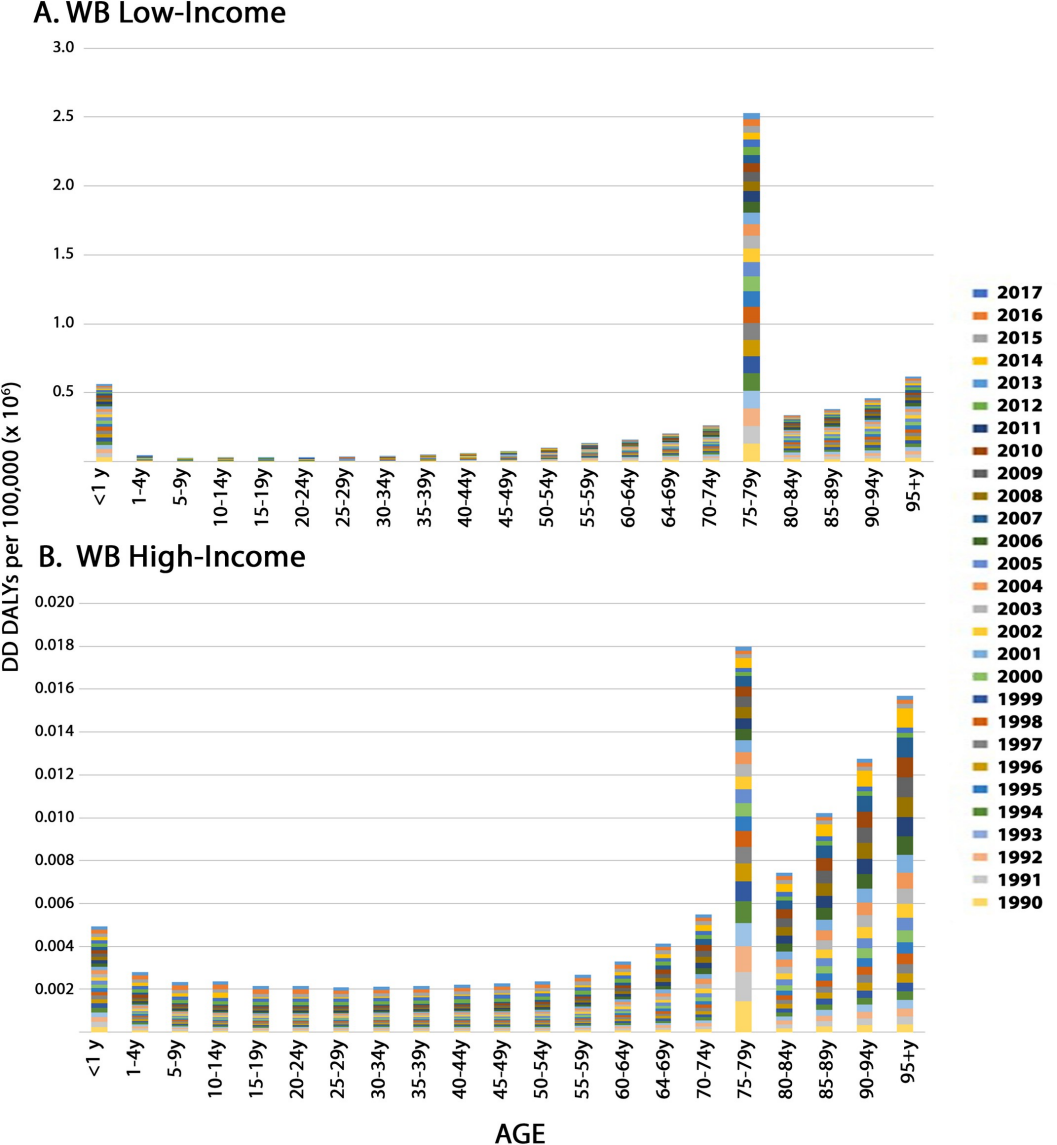
The relationship between age-standardized DD DALY rates per 100,000 and age. Age is given in years (y). The data are plotted for WB low income (A) and WB high-income (B) countries. For each age class (bar), the sum of annual DD DALY rates per 100,000 from 1990–2017 are presented.

To gain further insight into the age-related vulnerability to DD, we examined DD DALY rates (per 100,000) as a function of age (Fig 9). Interestingly, when considering population-standardized DD DALY rates, seniors over the age of 75 years seemed to be the most vulnerable to DD in both WB low-income (Fig 9A) and WB high-income (Fig 9B) countries.

## Discussion

In summary, we have examined the impact of DD over a 28-year period by examining DD DALYs, a measure that reports the number of lost “healthy” years in a population due to a specific illness. Overall, our analyses show that global DD DALYs have decreased dramatically by 85.45%. Interestingly, the decline in DD DALYs per 100,000 was more dramatic for WB low-income regions than for WB high-income regions. The temporal decrease in DD DALY rates in WB low-income countries was likely driven by a decrease in YLL. Alternatively, temporal increases in both YLL and YLD may have contributed to the apparent lack of progress in WB high-income countries. Incidence and prevalence rates have also decreased, albeit less dramatically than DD DALY rates. We also observed that the rate of change of DD DALYs was significantly correlated with SDI at a global scale (R^2^ = 0.993) as well as in WB low-income (R^2^ = 0.972) and WB high-income (R^2^ = 0.929) regions. When considering total DD DALYs, children under the age of 5 were the most vulnerable to DD regardless of WB income status. Furthermore, total DD DALYs were also increased for those of child-bearing years and the elderly. Interestingly, when considering DD DALY rates per 100,000, seniors over the age of 75 exhibited the highest vulnerability. In most years between 1990 and 2017, standardized DD DALYs for females exceeded those for males in WB high-income regions but were lower than those for males in WB low-income regions regardless of WB income class. To our knowledge, our study is the first to carry out a multi-year examination of DD DALYs associated with DD.

It is well known that DD disproportionately affect populations in poverty and conflict-ridden countries [3]. It was, therefore, unsurprising that we found a strong negative correlation between SDI and DD DALYs. In general, caregiver knowledge, as it relates to Water, Sanitation, and Hygiene (WASH), is an important factor in preventing diarrhea [47–49]. As the SDI value for a country increases so does the education level of its population, notably its women. Since women are the primary caregivers in most cultures, their education on WASH leads to a significant decrease in diarrheal rates among children [47–49]. We observed that the impact of SDI on DD weakens as a region’s SDI value increases. This was evident when we plotted DD DALYs as a function of SDI for WB high-income regions. The shape of the curve was statistically different and the rate of change (i.e., volatility) was the smallest for high-income regions. In other words, the relationship between SDI values and DD DALYs was the flattest.

Among WB low-income countries, Niger experienced the highest improvement in DD DALY rates over the 28-year period. This improvement may be the result of significant reductions in poverty achieved in this nation over the past decade [50]. On the other hand, an increased burden was observed for Central African Republic, which may be the result of civil strife, which has been occurring in this country from 2012 to the present [51, 52]. For all years from 1990–2017, North Korea had the lowest number of DD DALYs per 100,000. However, data from North Korea may be inaccurate or incomplete as information about disease in North Korea, including infectious diseases, is not widely available nor has it been broadly analyzed [53]. The WHO ranks available raw data from North Korea at the lowest level of credibility [53, 54].

Our analysis showed that cumulative DD DALY rates have not changed markedly in WB high-income regions over the past 28 years. There was a slight decrease from 1990 to 1998, after which DD DALY rates exhibit an annual increase for unclear reasons. This may reflect the already markedly low levels of DD DALYs in WB high-income countries, leaving little room for improvement. Progress may have been dampened by countries that exhibited little change over the study period (Antigua and Barbuda, Chile, Barbados, Estonia, Trinidad and Tobago) and by countries that exhibited average increases in nearly every year over the 28-year timeframe (Denmark, Germany, US Territories of Puerto Rico, Northern Mariana Islands, Guam). Guam exhibited the highest average annual increase in DD DALYs over the 28-year period (+5.67% per year). It is not known why the US territory of Guam has experienced these increases in standardized DD DALYs, or why it exhibits the highest standardized DD DALYs of all WB high-income constituents in 2017. This observation is supported by the IHME country report, which states that DD ranks 7^th^ as a leading cause of disability in Guam in 2017, which is an increase from its rank (9^th^) in 2007 [31].

The apparent stalled progress in WB high-income countries, may also indicate that the primacy of combating DD is decreasing. In support of this, Bump *et al*. [55] used 4 independent measures (trends in treatment coverage, changes in financial support, changes in perceived priority among experts, publication trends) to conclude the global priority to tackle DD has dropped to at least one-sixth of its level in the 1980s. Their data show that the priority to combat DD was displaced by priority given to malaria, tuberculosis, and HIV/AIDs, even though in 2017 DD claimed 1,569,600 lives, including 533,000 lives of children under the age of 5. These childhood mortality numbers are approximately 1.5 times the child mortality due to malaria, (354,300), 6.9 times the child mortality due to HIV/AIDs (77,500) and 9.3 times the child mortality due to tuberculosis (57,400) [31].

DALYs are comprised of both YLL and YLD measures. In WB low-income regions YLL exhibited a progressive decline, while YLD exhibited a progressive increase over the study period. Since an increasing YLD would signify an increasing DD burden, the overall decline in DD DALYs in WB low-income countries must be driven by the decrease in YLL. Alternatively, in WB high-income countries there were increases in both YLL and YLD over the study period. Therefore, the apparent lack of progress in WB high-income DD DALYs, is driven by an apparent lack of progress in both YLL and YLD numbers in these countries.

DALYs for individual etiologies of DD are not readily available. However, a recent study presented the global mortality statistics for 13 DD etiological agents, which are most significantly associated with moderate-to-severe diarrhea [56]. These agents included viruses (rotavirus, adenovirus, norovirus), bacteria (*Shigella*, *Vibrio cholera*, *Campylobacter*, non-typhoidal *Salmonella*, enterotoxigenic *E*. *coli*, enteropathogenic *E*. *coli*, *Aeromonas*, *Clostridium difficile*) and protists (*Cryptosporidium*, *Entamoeba*). For children under the age of five, bacterial infections seem to be the major cause of DD mortality, collectively accounting for ~49% of attributable deaths [56]. This is followed by viral infections, responsible for ~40% of attributable deaths, and protist infections, responsible for ~11% of attributable deaths [56]. Although all bacterial infections combined are responsible for the greatest number of DD-associated deaths in children under the age of 5, it is a viral agent, namely rotavirus, which is the single most deadly pathogen for this age group. Rotavirus is responsible for ~27% of total DD-associated deaths in children under five [56].

For individuals over the age of 70, bacterial infections are also the major cause of DD mortality, accounting for ~62.2% of attributable deaths [56]. Viral infections are responsible for ~33% of attributable deaths, while protist infections are responsible for ~4.8% of attributable deaths [56]. However, in the 70+ age class, a bacterium, *Shigella*, is the single deadliest pathogen, responsible for ~30% of total DD-associated deaths [56]. Given that different pathogens contribute uniquely to mortality in each of these two vulnerable age classes, strategies for managing DD must differ depending on age division. For example, increased deployment of rotavirus vaccine may assist in the control of DD in children under the age of five, while better emphasis on WASH interventions may ameliorate DD in the senior patient class.

Although DD-associated mortality in children under 5 years of age is high [20, 46], mortality rates have declined from 1990 to 2017 [57]. This progress in lowering childhood mortality is due to several factors including, female education (which has contributed to improvements in hygiene and sanitation), wider access to better nutrition, increased breastfeeding, better supplemental feeding, increased use of oral rehydration therapy (ORT), and measles immunization [58–60]. Several studies have shown that improvements in children’s nutrition, access to clean safe water and food, and treatment with rehydration therapy, have led to a reduction in the number of DD-related deaths [20, 61].

The substantial number of DD DALYs in WB low-income countries is alarming, particularly for children under five. Children who experience multiple bouts of DD start out on unequal footing for a quality life and experience less economic growth as adults [62]. The impact of DD on crucial early cognitive development is well-documented. Children who experienced multiple episodes of diarrheal have lower scores on the Test of Non-Verbal Intelligence-III [23] and the Wechsler Intelligence Scale for Children [22]. They also suffer visual-motor coordination impairment, slowed auditory short-term memory and information processing. These children are also at a higher risk of malnutrition, diabetes, and cardiovascular disease in adulthood [22, 24, 26, 63, 64].

Furthermore, DD have been linked to growth stunting in children [26, 63, 65]. An analysis of nine separate studies, carried out over a 20-year period in five countries, showed that stunting was directly linked to multiple episodes of diarrhea in infants under two years of age, where the proportion of stunting attributed to five or more episodes of diarrheal before 24 months increased the probability of stunting by 25% (95% CI 8–38%) [65]. The same study also showed that the likelihood of stunting was increased by 18% (95% CI 1–31%) for children who suffered from DD at least 2% of the time before 24 months of life. Finally, data show that children who experience stunting and recurrent gut infections are at increased risk of developing obesity and metabolic and cardiovascular comorbidities [26]. While the causes of these relationships are not fully understood, it has been postulated that nutrient deprivation, as a result of frequent gut infections, may cause epigenetic changes in gene expression regulating metabolism [26]. In support of this, a similar correlation between low birth weight and future risk of cardiovascular disease has been observed [66].

An increase in DD DALYs was also observed in young adults at 20 to 24 years of age in WB low-income countries and at 30 to 34 years of age in WB high-income countries. DD DALYs in this age group have not been extensively examined. Our data also demonstrate that seniors aged 60 to 64 years in WB low-income countries and 70 to 89 years in WB high-income countries were also substantially vulnerable to DD. That the peak of vulnerability occurs for an older group in WB high-income countries is likely a reflection of the increased lifespan of individuals in these regions. Previous studies have reported that diarrhea impacts at least 9% of the elderly population at any given time [67]. This has a significant negative impact on their quality of life, increasing their hospital visits [68]. Other studies have reported that DD in the elderly population are a significant cause of fecal incontinence [69], morbidity [70, 71], and mortality [72]. Long-term care facility stays for the elderly were cited as a risk factor for DD [72]. Consumption of multiple medications was also reported as a risk factor for DD, with an 11.0% increase of DD prevalence in patients who were taking 3–5 drugs, and an 11.7% increase in patients taking 6 or more drugs at once [73].

To our knowledge, this study is the first to carry out a multi-year examination of DALYs associated with DD. The GBD 2017 data set provides statistically robust morbidity assessments and both the strengths and limitations of GBD data are discussed elsewhere [74]. As for other studies that make use of global health data, our analysis has several limitations that may reduce the strength of our conclusions. First, surveillance capacity and awareness have undoubtedly changed over the 28-year data collection period. A recent comprehensive report [75] describes the timeline and evolution of the WHO Program for the Control of Diarrheal Diseases (CDD) from 1978 to 2015. The activities of the CDD have included the development and distribution of face-to-face and distance learning educational materials, and the support and communication of research on oral rehydration solution formulations, antidiarrheal drugs, antibiotics, and preventative measures. Although the upward trend in DD DALYs in WB high-income countries after 1998 may reflect an improvement in diagnostic capabilities, multiplexed molecular panels for the diagnosis of gastrointestinal infections have only been adopted in the last five years (reviewed in [76, 77]). Second, WB income group classifications, for all 28 years of data, are not publicly available. Therefore, we could not account for changes in the composition of such categories over the years. For this reason, we chose only high-income countries and low-income countries, as there would likely be significant movement of countries between categories in the middle-income ranges. Other studies that also compare data for WB income group have also used this approach (e.g., [78]). Third, since SDI and time are correlated, there is the possibility of over stating the role of SDI as a driver of DD DALYs rates. Indeed, other temporally changing factors, such as climate change, which are not necessarily correlated to SDI, may contribute to the observed changes in DD DALY trends.

While the world has seen marked decreases in DD mortality, much work is needed to reduce the global burden, especially for populations that are most at risk including children under five, the elderly, and caregivers. Overall, our analyses show that temporal reductions in DD DALYs are not equivalent across regions, sexes and age groups. Therefore, careful attention to local and demography-specific risk factors will be necessary to tailor solutions in region- and demography-specific manners.

## Supporting information

S1 Fig Age-standardized DD DALY rates from 1990 to 2017 for females and males in WB high-income (A) and WB low-income (B) constituents. Data for females presented in pink. Data for males presented in blue.
(TIF)

S2 Fig Comparison of standardized DD DALYs for males and females at the beginning of GBD data collection (1990) and at the end of data collection (2017) in WB high-income nations. Data for females presented in blue and grey. Data for males presented in orange and yellow. Arrowheads indicate the highest DD DALY rates for both males and females in 1990 (Saudi Arabia) or in 2017 (Guam).
(TIF)

S3 Fig Comparison of standardized DD DALYs for males and females at the beginning of GBD data collection (1990) and at the end of data collection (2017) in WB low-income nations. Data for females presented in blue and grey. Data for males presented in orange and yellow. Arrowheads indicate the highest DD DALY rates for both males and females in 1990 (Niger) or in 2017 for males (Central African Republic) or females (Chad).
(TIF)

S1 Table Summary of diarrheal disease DALY, incidence, and prevalence and global SDI values (1990–2017)^a^. (DOCX)
