# Supplementary figures and images for: Global estimated Disability-Adjusted Life-Years (DALYs) of diarrheal diseases: A systematic analysis of data from 28 years of the global burden of disease study
Source: PLoS One. 2021 Oct 27;16(10):e0259077. doi: 10.1371/journal.pone.0259077 (PMC8550424; doi:10.1371/journal.pone.0259077)

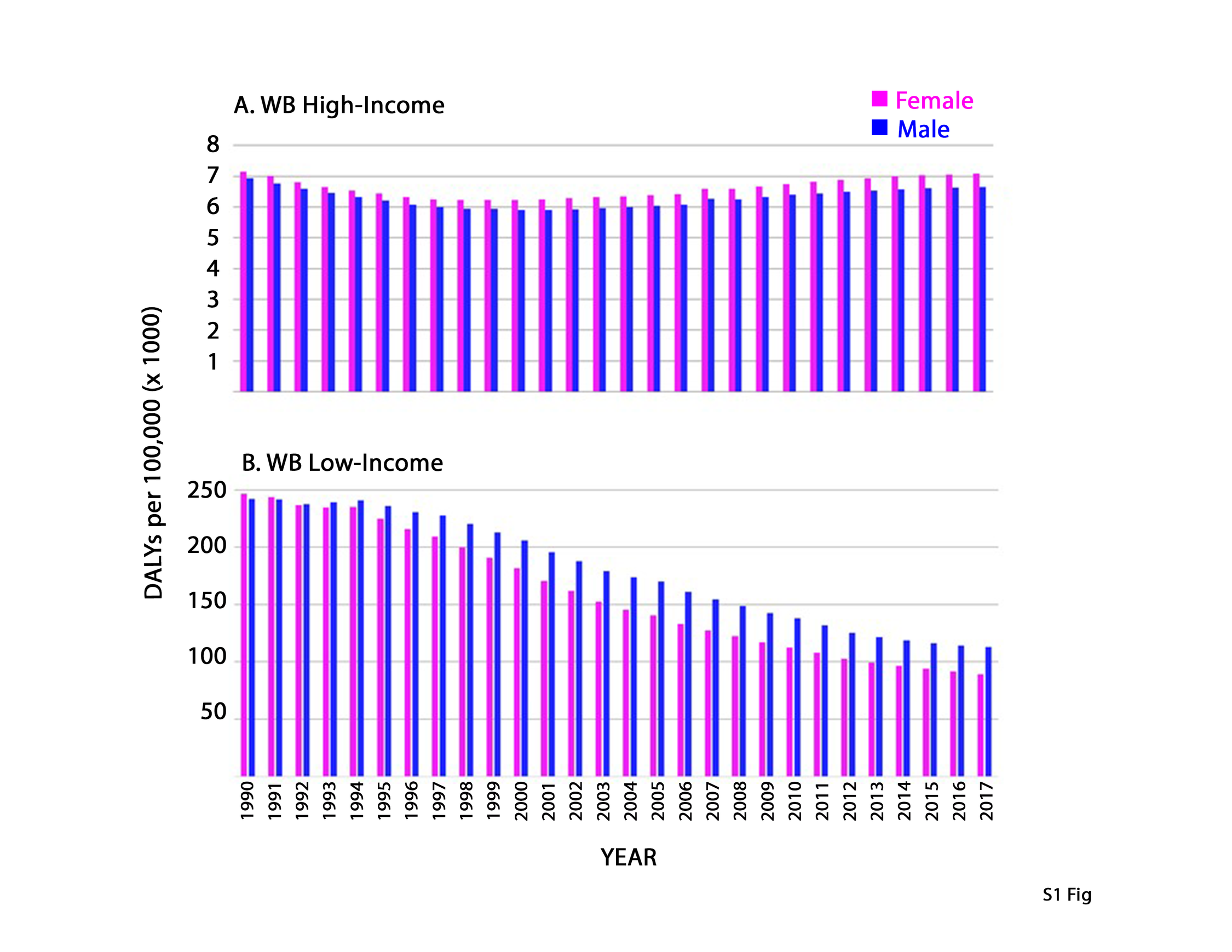

Supplement: S1 Fig — Age-standardized DD DALY rates from 1990 to 2017 for females and males in WB high-income (A) and WB low-income (B) constituents. Data for females presented in pink. Data for males presented in blue. (TIF) [file pone.0259077.s001.tif]

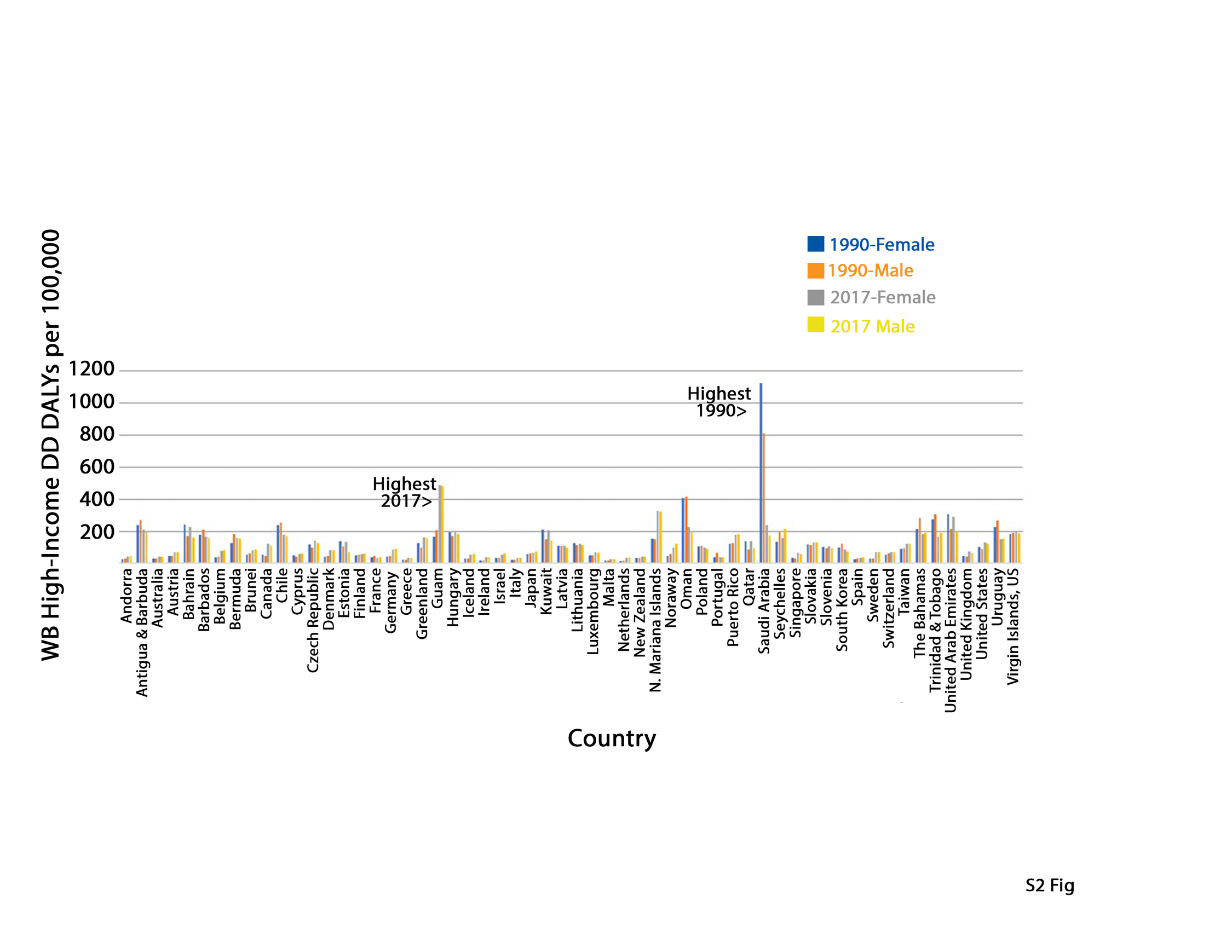

Supplement: S2 Fig — Data for females presented in blue and grey. Data for males presented in orange and yellow. Arrowheads indicate the highest DD DALY rates for both males and females in 1990 (Saudi Arabia) or in 2017 (Guam). (TIF) [file pone.0259077.s002.tif]

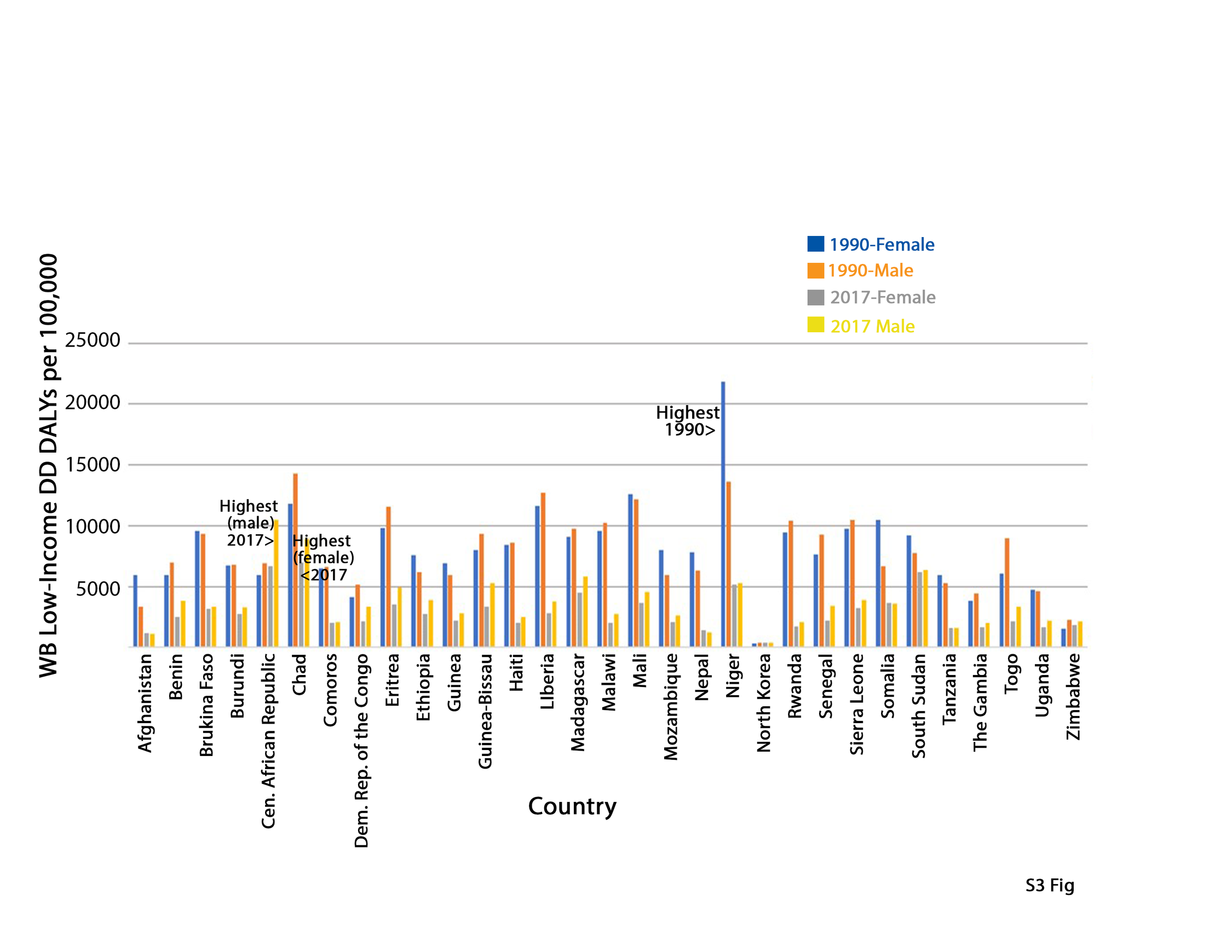

Supplement: S3 Fig — Data for females presented in blue and grey. Data for males presented in orange and yellow. Arrowheads indicate the highest DD DALY rates for both males and females in 1990 (Niger) or in 2017 for males (Central African Republic) or females (Chad). (TIF) [file pone.0259077.s003.tif]
