# Supplementary material for: Global estimated Disability-Adjusted Life-Years (DALYs) of diarrheal diseases: A systematic analysis of data from 28 years of the global burden of disease study
Source: PLoS One. 2021 Oct 27;16(10):e0259077. doi: 10.1371/journal.pone.0259077 (PMC8550424; doi:10.1371/journal.pone.0259077)
Supplement: S1 Table — (DOCX) [file pone.0259077.s004.docx]

S1 Table. Summary of Diarrheal Disease DALY, Incidence, and Prevalence and Global SDI Values (1990-2017)^a^

| Year | DALYs  Count (95% CI^b^) | DALYS  Rate per 100,000  (95% CI^b^) | DALYs APC^c^ | Incidence Count (95% CI^b^) | Incidence Rate per 100,000 (95% CI^b^) | Incidence APC^c^ | Prevalence Count  (95% CI^b^) | Prevalence Rate per 100,000 (95% CI^b^) | Prevalence  APC^c^ | SDI value |
| --- | --- | --- | --- | --- | --- | --- | --- | --- | --- | --- |
| 1990 | 178,669,278.768251  (154,235,572.289659- 203,868,117.467447) | 3,227.011526  (2,592.9728-3,853.0313) |  | 4,135,836,824.13  (3,811,339,413.89-4,500,759,366.83) | 85,808.8262  (78,417.423-93,386.464 |  | 63,492,658.0347 (59,217,728.4013-68,111,097.4995) | 1,333.944262  (1,232.43-1,438.42) |  | 0.52 |
| 1991 | 176,211,231.975992  (152,417,710.105072- 200,713,544.810044) | 3,119.762275  (2,504.7414-3,750.5701) | -3.32% | 4,178,680,590.28  (3,862,369,247.75-4546537194) | 85,243.90577  (78,179.059-92,445.771) | -0.66% | 63,879,563.7307 (59,687,506.0553-68,353,071.6295) | 1,312.280267  (1,216.89-1,409.32 | -1.62% | 0.53 |
| 1992 | 171,391,104.702396  (148,048,548.745833 -195,423,133.839874) | 3,196.685322  (2,571.7402-3,817.8921) | 2.47% | 4,226,896,542.33  (3,911,282,047.24 -4,594,888,290.47) | 84,750.95782  (77,939.813-91,774.906) | -0.58% | 64,354,761.6535 (60,216,614.2833-68,748,674.8797) | 1,322.448808  (1,223.84-1,422.15) | 0.77% | 0.53 |
| 1993 | 167,336,245.367074  (144,492,458.9642 -190,729,559.825914) | 2,957.94037  (,2390.7976-3,539.8182) | -7.47% | 4,278,217,512.01 (3,963,190,862.66-4,641,663,372.13) | 84,367.02074  (77,800.787-91,280.801) | -0.45% | 64,893,253.9959 (60,866,563.3427-69,276,193.9801) | 1,304.076211  (1,212.51-1,400.72) | -1.38% | 0.54 |
| 1994 | 163,531,094.361715  (141,140,449.762872- 185,920,622.828311) | 3,027.177112  (2,431.1143-3,628.4847) | 2.34% | 4,335,761,075.1 (4,021,226,056.65-4,701,326,266.54) | 84,131.51936  (90,943.807-77,592.173) | -0.28% | 65,553,334.1273 (61,573,352.4542-69,964,587.0143) | 1,296.138568  (1,210.38-1,391.45) | -0.61% | 0.54 |
| 1995 | 15,9309,627.496529  (137,848,574.959731- 181,558,619.24657) | 2,723.501965  (2,193.3654-3,289.062) | -10.03% | 4,402,215,884.49 (4,085,555,694.08-4,772,086,653.91 | 84,074.99025  (77,628.141-90,850.069) | -0.07% | 66,386,638.7427 (62,355,381.3153-70,879,899.7971) | 1,298.517448  (1,209.67-1,393.42) | 0.18% | 0.55 |
| 1996 | 153,873,908.661665  (133,776,006.168749-174,777,925.406401) | 2,789.759704  (2,253.8544-3,337.8316) | 2.43% | 4,484,297,876.71 (4,164,814,142.09-4,861,829,301.19) | 84,166.45238 (77,785.99-90,935.411) | 0.11% | 67,510,156.653 (63,422,181.5192-72,023,108.5209) | 1,296.420034  (1,211.28-1,390.79) | -0.16% | 0.55 |
| 1997 | 149,364,201.504314  (130,008,501.298731-170,059,247.258606) | 2,869.12157  (2,300.8089-3,439.5053) | 2.84% | 4584853195.09 (4261775119.74-4967966042.32) | 84,325.82129  (77,941.176-91,115.916) | 0.19% | 68,956,798.0921 (64,934,080.6913-73,382,360.6438) | 1,297.907349  (1,213.57-1,390.52) | 0.11% | 0.56 |
| 1998 | 145,736,711.34143  (127,033,666.155559-166,849,183.410077) | 2589.111841  (2104.2761-3128.5258) | -9.76% | 4,692,265,083.71 (4,359,523,454.04-5,081,709,755.37) | 84,520.07955  (78,208.538-91,259.493) | 0.23% | 70,525,663.0511 (66,426,872.4097-75,016,339.162) | 1,303.188161  (1,219.04-1,392.56) | 0.41% | 0.56 |
| 1999 | 141,526,595.809072  (123,282,039.652002-161,962,088.413046) | 2,662.658085  (2,158.4987-3,210.8337) | 2.84% | 4,795,836,579.09 (4,454,579,472.37-5,200,096,430.8) | 84,722.62697 (78,370.055-91,489.697) | 0.24% | 72,025,514.4601 (67,790,211.6672-76,472,410.1727) | 1301.905621  (1217.9-1391.17) | -0.10% | 0.57 |
| 2000 | 137,477,671.468813  (119,619,790.521677- 156,919,816.875706) | 2,414.347  (1,959.0251-2,894.9497) | -9.33% | 4,884,897,582.21 (4,542,372,128.38-5,299,281,709.2) | 84,897.61672  (78,560.554-91,696.696) | 0.21% | 73,265,259.8259 (68,959,087.3693-77,779,798.8537) | 1299.922729  (1215.52-1390.56) | -0.15% | 0.57 |
| 2001 | 132,617,150.351318  (115,367,283.211441-151,733,021.850802) | 2,503.494659  (2,035.3557-3,028.1257) | 3.69% | 4,943,837,569.91 (4,595,053,282.45-5,357,286,521.92) | 84,877.36704  (78,604.1-91,629.157) | -0.02% | 73,965,543.9256 (69,694,539.9109-78,441,687.4816) | 1,300.141976  (1217.16-1,388.9) | 0.02% | 0.58 |
| 2002 | 127,817,914.757423  (11,2012,414.876379-147,229,894.07108) | 2,322.244665  (1,900.9688-2,773.5586) | -7.24% | 4,973,994,368.72 (4,629,036,494.45-5,384,716,718.55) | 84,576.3535  (78,389.539-91,278.048) | -0.35% | 74,155,996.0217 (70,002,507.3735-78,620,590.4761) | 1,265.482821  (1,185.55-1,351.75) | -2.67% | 0.58 |
| 2003 | 123,279,674.410709  (108,332,371.69601-142,005,129.481731) | 2,134.52449  (1,784.9042-2,557.2886) | -8.08% | 4,988,840,828.18 (4648432090.04-5396015933.82) | 84,088.81068  (77,901.174-90,779.812) | -0.58% | 74,090,120.0219 (69,913,356.1542-78,561,557.6038) | 1,291.16644  (1,209.07-1,379.31) | 2.03% | 0.59 |
| 2004 | 119,766,167.975616  (105,447,221.525499-138,373,762.376267) | 1,936.484653  (1,629.9141-2,299.6167) | -9.28% | 5,001,354,629.58 (4,663,710,243.92-5,404,735,239.07) | 83,509.91353  (77,359.308-90,277.912) | -0.69% | 74,019,231.9529 (69,870,184.9237-78,475,500.0168) | 1,278.759649  (1,197.99-1,366.36) | -0.96% | 0.59 |
| 2005 | 116,788,626.814903  (102,742,156.942925-135,020,314.924957) | 2,227.685226  (1,844.292-2,659.984) | 15.04% | 5,028,649,926.53 (4,687,626,674.81-5,428,411,395.44) | 82,933.50831  (76,900.888-89,779.564) | -0.69% | 74,262,821.7028 (70,088,278.3286-78,729,936.5388) | 1,253.91104  (1,173.69-1,339.09) | -1.94% | 0.6 |
| 2006 | 114,876,510.325664  (101,308,896.047755-132,379,856.554444) | 2,062.667404  (1,727.0393-2,461.0739) | -7.41% | 5,052,841,968.73 (4,720,587,233.92-5,449,491,247.4) | 82,084.32847  (76,061.568-88,864.77) | -1.02% | 74,605,526.1742 (70,395,278.2749 -79,043,902.6142) | 1,203.463466  (1,127.73-1,284.27) | -4.02% | 0.6 |
| 2007 | 112,212,485.901006  (98,809,042.0435955-130,286,506.168655) | 2,009.495779  (1,695.3107-2,404.8831) | -2.58% | 5,059,634,422.65 (4,728,264,918.95-5,447,581,637.74) | 80,842.25968  (74,972.249-87,494.433 | -1.51% | 74,802,209.0738 (70,621,050.1784-79,117,542.0882 | 1,221.840846  (1,143.88-1,303.83) | 1.53% | 0.61 |
| 2008 | 110,181,306.352133  (96,998,856.4587667-127941188.924977) | 1,786.845807  (1,512.9897-2,121.3806) | -11.08% | 5,068,828,295.18 (4738222250.22-5452172271.14) | 79,561.16974  (73,801.076-86,030.035) | -1.58% | 75,088,786.9012 (70,899,515.0868-79,305,348.9356) | 1,240.235695  (1,161.03-1,323.88) | 1.51% | 0.61 |
| 2009 | 108,824,183.682178  (95,897,049.0614464-126,426,141.106932) | 1,865.834472  (1,582.0766-2,228.429) | 4.42% | 5099126336.86 (4,767,508,341.72-5,485,887,189.72) | 78,576.45609  (72,934.548-84,888.161) | -1.24% | 75,678,290.6528 (71,431,928.8358-79,819,137.9986) | 1,189.542034 (1,115.31-1,269.35) | -4.09% | 0.62 |
| 2010 | 107,018,962.602547  (93,454,209.9631604-124,889,985.741479) | 1,648.114883  (1,398.5254-1,943.8019) | -11.67% | 5,169,542,216.71 (4,833,451,585.31-5,556,928,325.15) | 78,222.35018  (72,627.136-84,517.125) | -0.45% | 76,789,507.0786 (72,456,555.2358-81,053,303.3758) | 1,194.46604  (1,118.08-1,275.49) | 0.41% | 0.62 |
| 2011 | 101,725,967.347824  (89,644,459.9831456-11,9068,365.99207) | 1,559.675946  (1,329.4942-1,851.3114) | -5.37% | 5,275,902,290.88 (4,920,054,852.45-5,665,447,574.68) | 78,498.33119  (72,855.167-84,815.449) | 0.35% | 78,374,946.7055 (73,913,067.4149-82,791,160.1963) | 1,184.499657  (1,110.83-1,264.35) | -0.83% | 0.62 |
| 2012 | 96,617,580.817008  (85,160,652.2047593-113,591,584.175086) | 1,712.459189  (1,446.174-2,021.7853) | 9.80% | 5,400,283,887.57 (5,025,690,557.65-5,801,308,983.15) | 79,061.51636  (73,334.703-85,468.255) | 0.72% | 80,228,490.5323 (75,541,509.5601-84,874,468.7924) | 1,187.770022  (1,112.96-1,267.93) | 0.28% | 0.63 |
| 2013 | 91843279.9738443  (80612660.6996005-108043934.094889) | 1,485.634218  (1,250.0568-1,771.3714) | -13.25% | 5,543,032,980.65 (5,152,024,164.31-5,964,838,486.52) | 79,794.94434  (73,946.876-86,323.494) | 0.93% | 82,356,346.493 (77,356,532.7585-87,174,559.0467) | 1,213.179482  (1,133.05-1,298.17) | 2.14% | 0.63 |
| 2014 | 87,650,670.122366  (76,585,397.9347087-103,537,964.791569) | 1,410.371267  (1,178.3664-1,687.8987) | -5.07% | 5,700,740,396.17 (5,288,676,893.47-6,146,170,370.65) | 80,558.81034  (74,583.265-87,152.899) | 0.96% | 84,706,121.3446 (79,257,785.5891-89,847,391.0056) | 1203.43125  (1125.25-1285.87) | -0.80% | 0.64 |
| 2015 | 83,908,966.8746052  (7,2856,397.2085521-99,796,525.7447586) | 1,288.01659  (1,059.3169-1,565.7738) | -8.68% | 5,870,845,742.81 (5,439,192,527.83-6,344,038,139.4) | 81,186.31937  (75,095.394-87,822.908) | 0.78% | 87,232,204.1209 (81,513,437.2364-92,749,672.126) | 1244.379661  (1159.52-1336.08) | 3.40% | 0.64 |
| 2016 | 81,339,102.2962044  (70,651,187.060819-97,118,555.3102732) | 1,343.444835  (1,110.2817-1,626.8633) | 4.30% | 6,069,024,218.27 (5,610,378,301.88-6,570,441,210) | 82,713.26257  (76,460.521-89,523.499) | 1.88% | 90,165,742.5137 (84,064,543.7691-96,111,684.5052) | 1,221.841335  (1,139.75-1,309.09) | -1.81% | 0.65 |
| 2017 | 81,039,363.8553969  (70,120,051.7904495-97,233,423.453799) | 1,262.767005  (1,030.1799-1,548.8008) | -6.01% | 6,292,936,671.74 (5,808,374,688.14-6,816,675,433.15) | 84,415.44027  (77,720.046-91,536.162) | 2.06% | 93,472,768.3654 (86,857,153.63-99,961,050.1615) | 1,269.775151  (1,180.95-1,364.57) | 3.92% | 0.65 |
|  | **Overall % Change**  **1990-2017** |  | **-85.43%** |  |  | **-1.53%** |  |  | **-4.45%** |  |

^a^Data from: Institute for Health Metrics and Evaluation (IHME). Findings from the Global Burden of Disease Study 2017. Seattle WA: IHME 2018 [31]

^b^95% confidence interval (95% CI). The upper and lower limits are presented.

^c^Annual Percent Change (APC)
